# Amorphous Silica Nanoparticles Obtained by Laser Ablation Induce Inflammatory Response in Human Lung Fibroblasts

**DOI:** 10.3390/ma12071026

**Published:** 2019-03-28

**Authors:** Sorina Nicoleta Voicu, Mihaela Balas, Miruna Silvia Stan, Bogdan Trică, Andreea Iren Serban, Loredana Stanca, Anca Hermenean, Anca Dinischiotu

**Affiliations:** 1 Department of Biochemistry and Molecular Biology, Faculty of Biology, University of Bucharest, 91-95 Splaiul Independentei, 050095 Bucharest, Romania; sori.petrache@yahoo.com (S.N.V.); radu_mihaella@yahoo.com (M.B.); miruna.stan@bio.unibuc.ro (M.S.S.); anca.hermenean@gmail.com (A.H.); 2 Department of Pharmacy, Faculty of Pharmacy, Titu Maiorescu University, 004051 Bucharest, Romania; 3 The National Institute for Research & Development in Chemistry and Petrochemistry (INCDCP-ICECHIM), 202 Splaiul Independentei, 060021 Bucharest, Romania; trica.bogdan@gmail.com; 4 Department of Preclinical Sciences, University of Agronomical Sciences and Veterinary Medicine, 105 Splaiul Independentei, 050097 Bucharest, Romania; irensro@yahoo.com (A.I.S.); lory_stanca@yahoo.com (L.S.); 5 Department of Experimental and Applied Biology, Institute of Life Sciences, Vasile Goldis Western University of Arad, 86 Rebreanu, 310414 Arad, Romania; 6 Department of Histology, Faculty of Medicine, Vasile Goldis Western, University of Arad, 1 Feleacului, 310396 Arad, Romania

**Keywords:** silica nanoparticles, MRC-5 cell line, oxidative stress, inflammatory response

## Abstract

Silica nanoparticles (SiO_2_ NPs) represent environmentally born nanomaterials that are used in multiple biomedical applications. Our aim was to study the amorphous SiO_2_ NP-induced inflammatory response in MRC-5 human lung fibroblasts up to 72 hours of exposure. The intracellular distribution of SiO_2_ NPs was measured by transmission electron microscopy (TEM). The lactate dehydrogenase (LDH) test was used for cellular viability evaluation. We have also investigated the lysosomes formation, protein expression of interleukins (IL-1β, IL-2, IL-6, IL-8, and IL-18), COX-2, Nrf2, TNF-α, and nitric oxide (NO) production. Our results showed that the level of lysosomes increased in time after exposure to the SiO_2_ NPs. The expressions of interleukins and COX-2 were upregulated, whereas the expressions and activities of MMP-2 and MMP-9 decreased in a time-dependent manner. Our findings demonstrated that the exposure of MRC-5 cells to 62.5 µg/mL of SiO_2_ NPs induced an inflammatory response.

## 1. Introduction

Nanoparticles (NPs) present special mechanical and physicochemical properties due to their high surface area/volume rate, which confer biological reactivity and toxicological potential because their size scale is comparable with that of biological macromolecules [1]. They are environmental born or engineered, and used in domestic, industrial, and medical activities. 

Natural silica (SiO_2_) is an important constituent of sand and quartz, and is present in natural composites, such as those of *Rossella fibulata* sponge [2]. Natural SiO_2_ is a base material for the glass, optical, and building industries.

There are two forms of SiO_2_ NPs—crystalline and amorphous—that have the same molecular formula, but different structural arrangements, i.e. regular for crystalline forms, and irregular for amorphous forms [3]. Amorphous SiO_2_ nanoparticles that are formed by laser ablation can represent a promising material for a variety of applications. These can be used as fillers in plastics, rubber, and tires in industry [4]; as thermal and electrical insulators, humidity sensors, electronic substrates, and enhancers of oil recovery; and in dye production [5]. In addition, they are used as food additives (E551), such as anticaking agents in spices and instant soups [6], and dental fillers [3]. Also, these kinds of particles are manufactured for diagnostic and biomedical research, because it is easy to obtain them at relatively low cost. Human exposure to SiO_2_ NPs can occur by dermal contact, inhalation, and ingestion [7]. Airborne particles are inhaled and their fate in the respiratory tract depends on the anatomic structure and aerodynamic characteristics of particles [8]. 

Unlike larger particles, the NPs can access the deep alveolar region where removal mechanisms are not completely efficient [9]. The particle clearance in the alveoli is achieved mainly by macrophages [10]. If nanoparticles have not been cleared by phagocytosis, they transmigrate the alveolar epithelium and enter the connective tissue of the lung [11], systemic circulation, and the lymph nodes [9]. 

In human subjects, occupational crystalline silica exposure induced silicosis [12], and it was proved to be carcinogenic [13]. In addition, rats exposed to aerosols containing crystalline SiO_2_ particles presented significant increases in telomere length, which can be associated with a high risk of lung cancer development [14]. The amorphous SiO_2_ NPs were considered less toxic than the crystalline ones, but emerging evidence from experimental studies has shown that they have similar toxic effects [15]. Analyzing an important amount of published data regarding the amorphous SiO_2_ NPs, Murugadoss et al. [16] could not conclude if these have exposure effects comparable with those generated by micrometric crystalline SiO_2_ [16]. Previously, it was proved that Si/SiO_2_ quantum dots triggered an inflammatory response in pulmonary fibroblasts [17], and acute short exposure to silica nanoparticles induced high levels of interleukins IL-5, IL-13, IL-1β, and IFN-γ [18]. Recently, Wu et al. [19] revealed that SiO_2_ NPs resulted in the reduction of viability of adenocarcinoma human alveolar basal epithelial A549 cells and the upregulation of IL-1β and IL-6 [19].

In vitro, silica nanoparticles induced inflammation in human umbilical vein endothelial cells (HUVEC), [20] human hepatoma cells (Huh 7) [21], as well as mouse J774A.1 macrophage cells [22]. On the other hand, a single instillation of amorphous silica NPs induced subchronic inflammatory responses and histological changes in mice lungs [23].

In this context, taking it into account that the inflammatory response in lung fibroblasts post-exposure to amorphous SiO_2_ NPs was less investigated, and inhalation is an important method of exposure, we aimed to study the modulation of some markers of inflammation and oxidative stress in the human lung fibroblast MRC-5 cells treated with these NPs.

## 2. Materials and Methods 

### 2.1. Chemicals and Reagents 

Fetal bovine serum (FBS) and modified Eagle’s medium (MEM) were purchased from Gibco (Grand Island, N.Y, USA), and phosphate-buffered saline (PBS), Western Breeze Chromogenic Kit anti-mouse, anti-rabbit, and anti-goat, and LysoTracker Green DND-26 were bought from Invitrogen (Carlsbad, CA, USA). Kit lactic dehydrogenase-based, sulfanilamide, H_3_PO_4_, and *N*-1-naphthylethylenediamine dihydrochloride were obtained from Sigma-Aldrich, St. Louis, MO, USA. Antibodies for IL-1β (H153): sc-7884, IL-2 (C2-1-hIL-2): sc-32295, IL-6 (E-4): sc-28343, IL-8 (807): sc-52870, IL-18 (H-173): sc-7954, tumor necrosis factor-alpha (TNF-α) (N-19): sc-1350, cyclooxygenase-2 (COX-2) (M-19): sc-1747, nuclear factor (erythroid-derived 2)-like 2 (Nrf-2) (H-300): sc-13032, matrix metalloproteinase-2 (MMP-2) (H-76): sc-10736, matrix metalloproteinase-9 (MMP-9) (M-17): sc-6841, nuclear factor kappa-light-chain-enhancer of activated B cells (NF-kB) p65 (A): sc-109 and β-actin (C4): sc-47778 were purchased from Santa Cruz Biotechnology (Santa Cruz, CA, USA) and Invitrogen (Carlsbad, CA, USA). All of the other chemicals used were of analytical grade and were purchased from Sigma (St. Louis, MO, USA). 

### 2.2. SiO_2_ NPs Preparation and Characterization

SiO_2_ NPs were obtained from NaBond Technologies Co., Ltd. (Shenzhen, China). The synthesis and characterization of NPs were described in detail in our previous paper [24]. Briefly, amorphous SiO_2_ NPs were synthesized by the reactive laser ablation method and homogenized in Milli-Q water, ultrasonicated, and sterilized by autoclaving at 121 °C for 15 to 20 min. The NPs are spherical in shape and have a mean size of less than 10 nm. The NPs characteristics were analyzed using transmission electron microscopy (TEM) (FEI, Hillsboro, OR, USA), energy-dispersive X-ray spectroscopy (EDX) (Oxford Instruments, Abingdon, UK), and zeta potential analyses. The SiO_2_ NPs were dispersed in distilled water at a concentration of 250 μg/mL. Three small drops (10 µL) of this diluted mixture were added consecutively on top of a pure carbon film copper grid (Ted Pella, Inc., Redding, CA, USA). Each time, the excess was carefully removed using filter paper. The sample was analyzed in bright field mode using a Tecnai F20 G^2^ TWIN crio- TEM from (FEI, Hillsboro, OR, USA) at an acceleration voltage of 200 kV. The zeta potential of SiO_2_ NPs (62.5 μg/mL) in ultrapure Milli-Q water was assessed using a Malvern Nano-ZS instrument (Malvern Instruments, Malvern, Worcestershire, UK). The measurements were performed in triplicate at 25 °C using the refractive index of 1.46.

### 2.3. Cell Culture and Treatment

The MRC-5 (CCL-171) cell line was provided by the American Type Culture Collection (ATCC), cultured in MEM supplemented with 10% heat-inactivated FBS, and maintained in a 95% humidified atmosphere (with 5% CO_2_) at 37 °C. The cells were harvested at 80% confluence using 0.25% trypsin and −0.03% ethylenediaminetetraacetic acid (EDTA) (Sigma-Aldrich, St. Louis, MO, USA) and sub-cultured into 75-cm^2^ flasks (90076, Techno Plastic Products (TPP), Trasadingen, Switzerland), six-well plates (92006, TPP, Trasadingen, Switzerland) or 24-well plates (92024, TPP, Trasadingen, Switzerland) according to the experiment types. Before treatment, the nanoparticles stock suspension (12.5 mg/mL) was sonicated for 45 minutes in an ultrasonic bath. Afterwards, the sterile SiO_2_ NPs suspension was added in culture medium at a final concentration of 62.5 µg/mL, and media and cells were collected after the three exposure intervals.

### 2.4. The Lysate Preparation 

After exposure intervals, the cells were detached by 0.25% trypsin and 0.03% EDTA solution, centrifuged, and re-suspended in PBS. The cell suspension was sonicated (three times, 30 seconds, on ice). The debris were removed by centrifugation at 5000 rpm, 10 min, at 4 °C. Finally, the supernatant was aliquoted and frozen at −80 °C, for subsequent biochemical and immunochemical analyses.

### 2.5. Protein Concentration Assessment 

Protein concentration was determined by the Bradford method [25] using bovine serum albumin as standard (Sigma-Aldrich, St. Louis, MO, USA). 

### 2.6. Electron Microscopy

For transmission electron microscopy (TEM), 1 × 10^6^ cells were exposed to SiO_2_ NPs. Post-treatment, cells were trypsinized and centrifuged (1200 rpm) at 24 h, 48 h, and 72 h. The pellets were prefixed in 2.7% glutaraldehyde in 0.1 M of phosphate buffer for 1.5 h at 4 °C, and washed in 0.15 M of phosphate buffer (pH 7.2). Post-fixation has been done in 2% (w/v) osmic acid in 0.15 M of sodium phosphate buffer at 4 °C for 1 h. Samples were dehydrated in acetone and included in epoxy embedding resin. Sections 70-nm thick were made on a Leica EM UC7 ultramicrotome (Leica microsystems GmbH, Wetzlar, Germany), doubly contrasted, and analyzed with a Tecnai 12 Biotwin transmission electron microscope (FEI Company, Eindhoven, The Netherlands). The panels displayed are representative of 50 panel views.

### 2.7. The Quantification of Lysosomes with Lyso Tracker Green DND-26

Briefly, cells attached overnight on coverslips were treated with 62.5 µg/mL of SiO_2_ NPs, and after each interval, the samples were prepared and analyzed on an Olympus IX71 inverted epifluorescence microscope (Olympus, Tokyo, Japan). Lysosomes were stained with 100 nM of LysoTracker Green DND-26 for 30 min at 37 °C and 5% CO_2_. Cells nuclei were stained with 2 µg/mL of 4, 6-diamino-2-phenylindole (DAPI) for 10 min at room temperature. The total fluorescence intensity was quantified using ImageJ, version 1.48 (National Institutes of Health, Bethesda, MD, USA), and expressed relative to control cells. 

### 2.8. Lactate Dehydrogenase (LDH) Release Assay

Briefly, MRC-5 cells were cultured in 24-well plates at a density of 5 × 10^4^ cells/well and incubated overnight at 37 °C and 5% CO_2_. After 12 h of growth, cells were treated with 62.5 µg/mL of SiO_2_ NPs for 24 h, 48 h, and 72 h. A volume of 50 μL cell medium was used for LDH activity measurement. The absorbance at 450 nm was measured with a microplate reader and the data obtained were expressed relative to control.

### 2.9. Western Blot Analysis

The samples containing 30 µg of protein were resolved by 10% or 12% sodium dodecyl sulfate polyacrylamide gel electrophoresis (SDS-PAGE) and transferred onto a 0.45 µm polyvinylidene difluoride membranes (PVDF) membrane in a wet transfer system, at 350 mA, for two hours, at 4 °C. Then, the membranes were treated with blocking solution for 30 min at room temperature, and incubated with appropriate diluted solutions of anti-IL-1β (1:250), anti-IL-6 (1:250), anti-IL-8 (1:250), anti-TNF-α (1:250), anti-COX-2 (1:250), anti-Nrf-2 (1:250), anti-NF-kB p65 (1:250), anti-MMP-2 (1:500), anti-MMP-9 (1:500), anti-IL-2 (1:250), anti-IL-18 (1:250), and anti-β-actin (1:1000) primary antibodies. Afterwards, the membranes were treated with anti-mouse, anti-rabbit, or anti-goat secondary antibodies coupled with alkaline phosphatase and 5-bromo-4-chloro-3-indolyl-phosphate / nitro-blue tetrazolium (BCIP/NBT) as a chromogenic substrate. The protein bands were visualized with ChemiDoc Imaging System (Bio-Rad, Hercules, CA, USA), quantified with Image Lab software ( version 5.2, Bio-Rad, Hercules, CA, USA), and normalized to the β-actin one. The results were represented as percentage related to control. 

### 2.10. Measurement of Nitric Oxide (NO) Concentration

The MRC-5 fibroblasts were seeded at a density of 5 × 10^5^ cells/ml culture medium and treated with 62.5 μg/mL of SiO_2_ NPs for 24 h, 48 h, and 72 h, respectively. After that, a volume of 80 µL of culture medium and 80 µL of Griess reagent (1% sulfanilamide and 0.1% N-(1-naphthyl)-ethylenediamine dihydrochloride in a ratio of 1:1) were incubated for 5 min at room temperature, in dark, according to the Griess method [26]. 

Absorbance was measured at 550 nm using a microplate reader Thermo Scientific Appliskan (Thermo Scientific, Rockford, IL, USA) and quantified using a calibration curve with sodium nitrite. 

### 2.11. SOD Zymography

Samples of 50 μg of protein were run on 15% PAGE at 90 V and 4 °C. The gels were stained for superoxide dismutase (SOD) activity using nitroblue tetrazolium, riboflavin, and tetrametyletylenediamine (TEMED) according to the Beauchamp and Fridovich method [27]. SOD activity was visualized using ChemiDoc Imaging System (Bio-Rad, Hercules, CA, USA) and the bands were quantified with Image Lab software (version 5-2, Bio-Rad, Hercules, CA, USA). 

### 2.12. Caspase-1 Activity Assay

The analysis of caspase-1 activity is based on the spectrophotometric detection of p-nitroaniline chromophore (pNA) after cleavage of the YVADpNA substrate. Briefly, MRC-5 cells were treated with SiO_2_ NPs for 24 h, 48 h, and 72 h, respectively. At a volume of 50 μL of cell lysate obtained after each exposure interval, an equal volume of reaction buffer containing dithiothreitol (DTT) was added. Then, 5 μL of substrate YVADpNA was added and incubated for 1 h at 37 ° C. After that, absorbance was read at 405 nm using a FlexStation 3 Multi-Mode Microplate Reader (Molecular Devices LLC, San Jose, CA, USA) plate reader. The results obtained were reported to control cells.

### 2.13. Gelatin Zymography

After treatment with SiO_2_ NPs, the supernatants (serum-free culture) were concentrated by centrifugation using Centricon filtration units (Amicon Ultra-15 Centrifugal Filters, 10,000 MWCO, cellulose, Merck Millipore, Tullagreen, Carrigtwohill, Cork, Ireland). The samples containing 50 µg of protein were separated on 10% SDS PAGE containing 0.2% gelatin and 0.03% casein, at 90 V and 4 °C, respectively. Then, gels were washed with distilled water and incubated twice for 15 min with 50 mM of Tris HCl pH 7.6 buffer containing 2.5% Triton X-100. Then, the gels were washed again with distilled water and incubated overnight at 37 °C with 50 mM of Tris HCl pH 7.6 buffer with 10 mM of CaCl_2_, 50 mM of NaCl, and 0.05% Brij 35. The gels were stained with Coomasie Brilliant Blue, and the enzymatic activities of metalloproteinases were noticed as white bands on the blue gels after incubation with destaining solution. The gels were scanned using ChemiDoc Imaging System (BioRad), and bands were quantified with Image Lab software (BioRad). 

### 2.14. Statistical Analysis 

The results were analyzed for statistical significance by Student’s *t* test (Microsoft Excel, Microsoft, Redmond, WA, USA) and presented as a percentage of the control values. Data are represented as mean ± standard deviation from three independent experiments. A value of *p* less than 0.05 was considered to be statistically significant.

## 3. Results

### 3.1. Nanoparticles Characterization 

**Figure 1:**
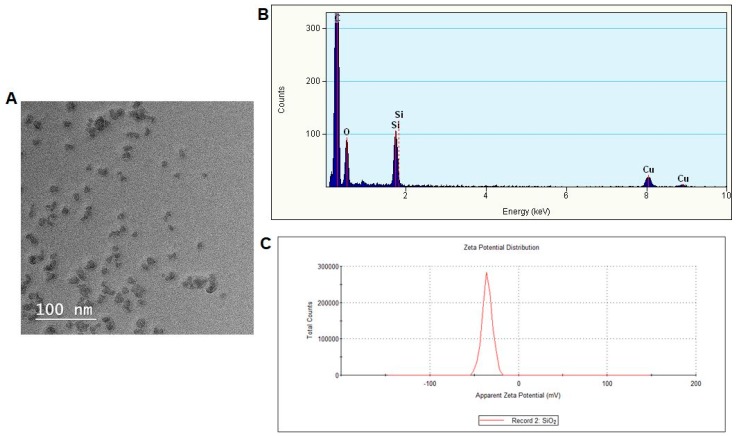
Characterization of SiO_2_ NPs by TEM (**A**), energy-dispersive X-ray spectroscopy (EDX) (**B**), and zeta potential (**C**) analyses.

TEM analysis showed that SiO_2_ NPs are very similar in size, measuring less than 10 nm in diameter (Figure 1A). However, their shape is variable. The nanoparticles tend to form aggregates between 50–500 nm, although this is probably due to the uncontrolled drying conditions in the TEM preparation method. The energy-dispersive X-ray spectroscopy (EDX) determination revealed the presence of silicon and oxygen in a proportion of 1:2, confirming the pure SiO_2_ composition (Figure 1B). The copper and carbon contents were derived from the carbon holey copper grids for TEM and used as substrates for this analysis. The analysis of zeta potential is a measure of the effective electric charge on the NP surface and can also be used to describe the adsorption mechanisms of biological ligands on the surface of NPs. The average value of zeta potential determined for SiO_2_ NPs was −35.4 ± 0.404 mV, which confirmed the electrostatic stabilization of particles dispersed in water (Figure 1C).

### 3.2. Ultrastructural Studies 

**Figure 2:**
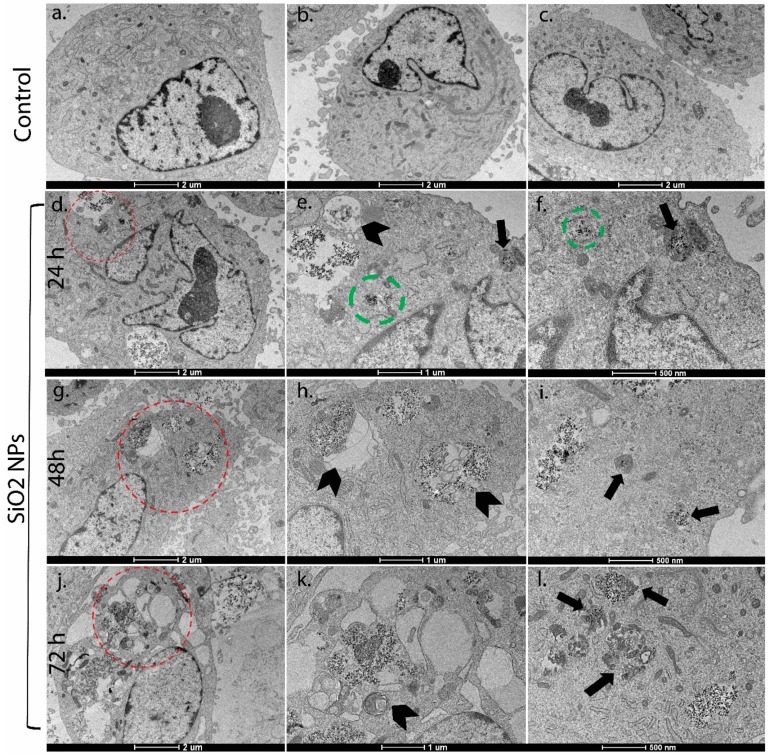
Transmission electron microscopy images of MRC-5 cells with SiO_2_ NPs treatment for 24 h (**a**), 48 h (**b**), and 72 h (**c**). Silica nanoparticles (SiO_2_ NPs) were not observed in untreated cell controls and the cellular structures were unchanged at 24 h, 48 h, and 72 h (**a**,**e**,**i**); Electron-dense SiO_2_ NPs were spread in cytoplasm, lysosomes, and autophagic vacuoles in SiO_2_ NP-treated cells, which increased with exposure time (**d**,**g**,**j**). The magnification view of the selected area (red circles) showed autophagic vacuoles (arrowheads) containing aggregated SiO_2_ NPs (**e**,**h**,**k**); Figures (**f**,**i**,**l**) illustrated SiO_2_ NPs-loaded lysosomes (arrows) and cytoplasmic SiO_2_ NPs (green circle). *Scale bars*: 2 μm (**a**–**d**,**g**,**j**); 1 μm (**e**,**h**,**k**); 500 nm (**f**,**i**,**l**). *Magnification*: (**a**–**d**,**g**,**j**) 6000×; (**e**,**h**,**k**) 11,500×; (**f**,**i**,**l**) 20,500×.

TEM analysis represents the best method to put in evidence the intracellular localization of NPs and the colocalization of NPs with lysosomes. MRC-5 cells treated with SiO_2_ NPs clearly manifested the prominent accumulation of SiO_2_ NPs within lumens of lysosomes (Figure 2f,i,l—arrows after 24 h, 48 h, and 72 h, respectively) and autophagic vacuoles (Figure 2e,h,k—arrowheads after 24 h, 48 h, and 72 h, respectively) in a time-dependent manner, compared to the untreated cells. Moreover, some SiO_2_ NPs were dissipated in cytoplasm, mainly after 24 h of exposure (Figure 2e,f—green circles). 

### 3.3. The Quantification of Lysosomes with Lyso Tracker Green in Human Lung Fibroblasts 

**Figure 3:**
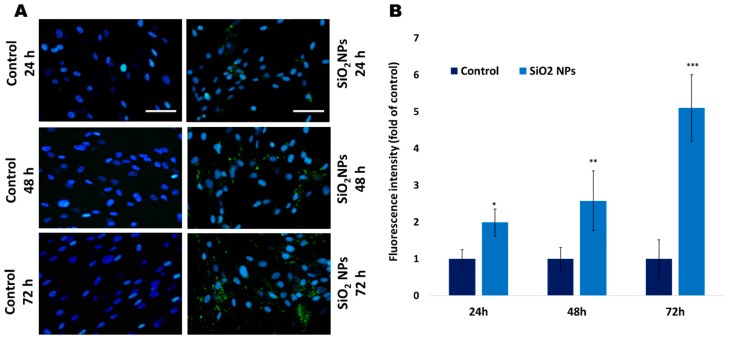
(**A**) Representative images showing green lysosomes stained with LysoTracker Green and blue nuclei stained with 4, 6-diamino-2-phenylindole in MRC-5 cells. Scale bars (50 µm) are similar for all images. (**B**) Quantification of lysosomes in MRC-5 cells. Values are mean SD (n = 3). Statistical analysis was assessed by Student’s *t* test (* *p* < 0.05, ** *p* < 0.01, and *** *p* < 0.001 compared to control).

The fluorescence microscopy images (Figure 3A) show an accumulation of lysosomes especially after 48 h and 72 h of exposure. In Figure 3B, it can be noticed that the fluorescence intensity was significantly increased by 1.58 and four times after 48 h and 72 h, respectively, compared to control. These results are in agreement with the TEM analysis shown in Figure 2f,i,l, confirming the colocalization of SiO_2_ NPs with lysosomes after 24 h, 48 h, and 72 h, respectively.

### 3.4. Lactate Dehydrogenase (LDH) in Human Lung Fibroblasts 

**Figure 4:**
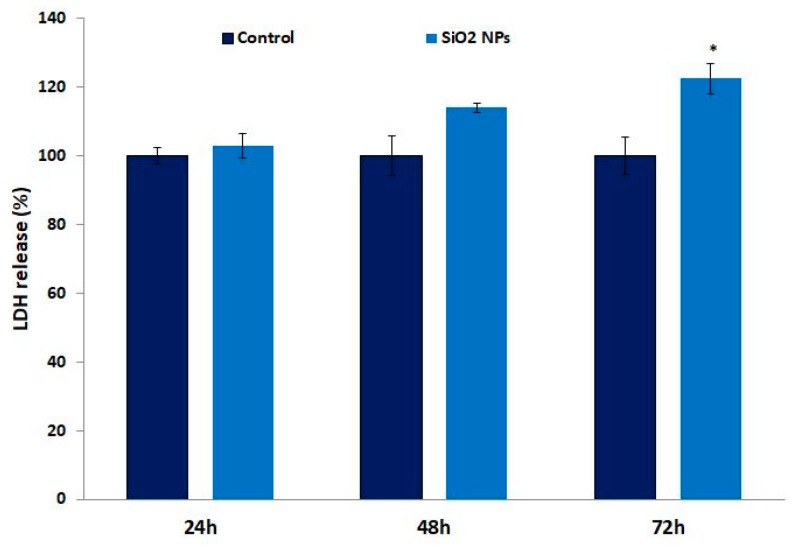
Lactate dehydrogenase (LDH) level in the culture medium of MRC-5 cells after exposure to SiO_2_ NPs for 24 h, 48 h, and 72 h. Values are mean SD (n = 3). * *p* < 0.05.

LDH is a cytosolic enzyme that can be released into the culture medium in case of plasma membrane damage. After 48 h and 72 h of exposure to SiO_2_ NPs, the level of LDH release increased by 14% and 22%, respectively, compared to control cells (Figure 4). 

### 3.5. Expression of Nrf-2, NF-kB p-65 Proteins, and Nitric Oxide (NO) Release in Human Lung Fibroblasts

**Figure 5:**
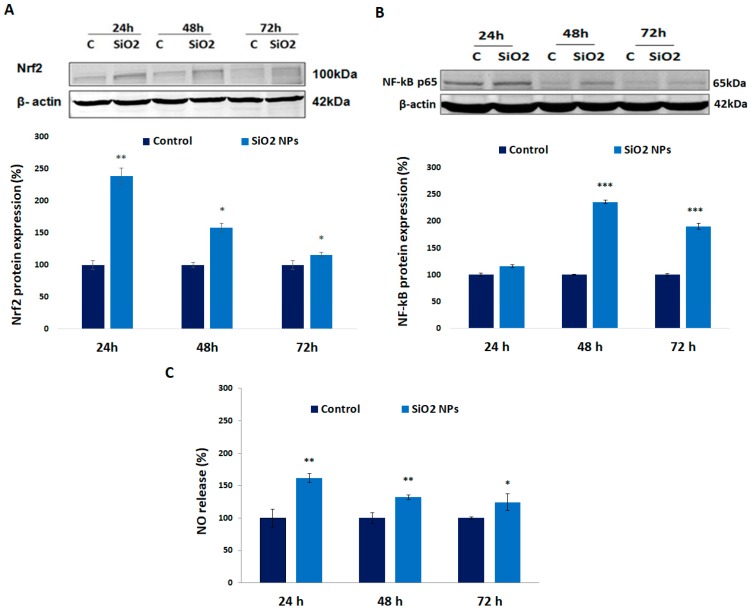
(**A**) Expression of nuclear factor (erythroid-derived 2)-like 2 (Nrf-2), (**B**) NF-kB proteins, and (**C**) nitric oxide release in MRC-5 cells after exposure to SiO_2_ NPs. Values are mean SD (n = 3). * *p* < 0.05, ** *p* < 0.01, and *** *p* < 0.001.

As it can be noticed in Figure 5A, the expression of protein Nrf-2 increased by 139%, 58%, and 16%, whereas the NF-kB p65 (Figure 5B) protein upregulated with 16%, 135%, and 90% compared to control after 24 h, 48 h, and 72 h, respectively. Also, nitric oxide concentration (Figure 5C) increased significantly by 62%, 32%, and 24% after 24 h, 48 h, and 72 h, respectively, of exposure.

### 3.6. The Enzymatic MnSOD and CuZn-SOD Activities in Human Lung Fibroblast 

**Figure 6:**
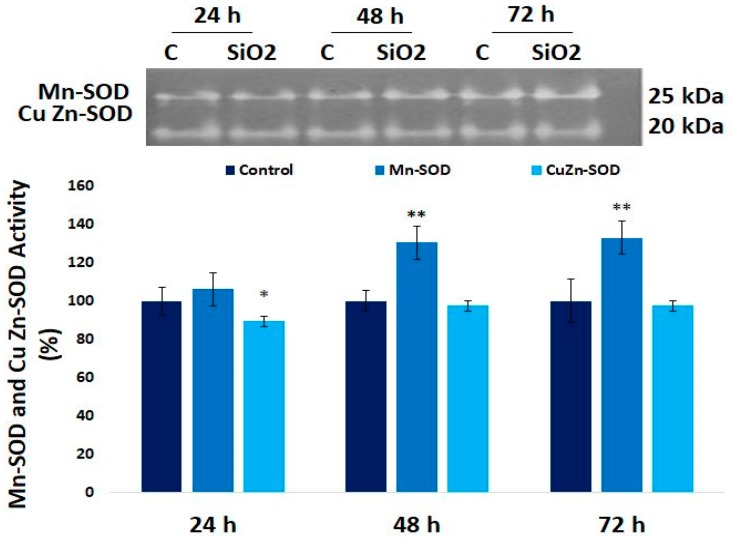
Zymographic analysis of MnSOD and CuZn-SOD after 24 h, 48 h, and 72 h of exposure at 62.5 μg/mL SiO_2_. Values are mean SD (n = 3). * *p* < 0.05, ** *p* < 0.01.

The MnSOD activity increased by 30% and 33% after 48 h and 72 h exposure compared to controls, whereas CuZn-SOD activity was diminished after the first 24 h, and remained at the level of control up to the end of treatment (Figure 6). 

### 3.7. Cyclooxygenase-2 (COX-2) and Tumor Necrosis Factor Alpha (TNF-α) Proteins in Human Lung Fibroblasts 

**Figure 7:**
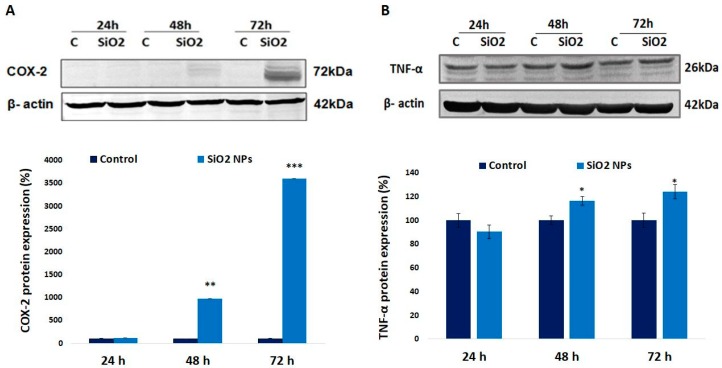
Expression of (**A**) cyclooxygenase-2 (COX-2) and (**B**) tumor necrosis factor-alpha (TNF-α) proteins after 24 h, 48 h, and 72 h. Values are mean SD (n = 3). * *p* < 0.05, ** *p* < 0.01, and *** *p* < 0.001.

The protein levels of COX-2 and TNF-α were upregulated in a time-dependent manner (Figure 7). After 72 h of exposure, the level of COX-2 significantly increased by about 3600%, whereas the TNF-α level increased only by 24% compared to control.

### 3.8. Expression of Interleukin (IL-1β, IL-2, IL-18, IL-6, and IL-8) and Caspase-1 Activity in Human Lung Fibroblasts

**Figure 8:**
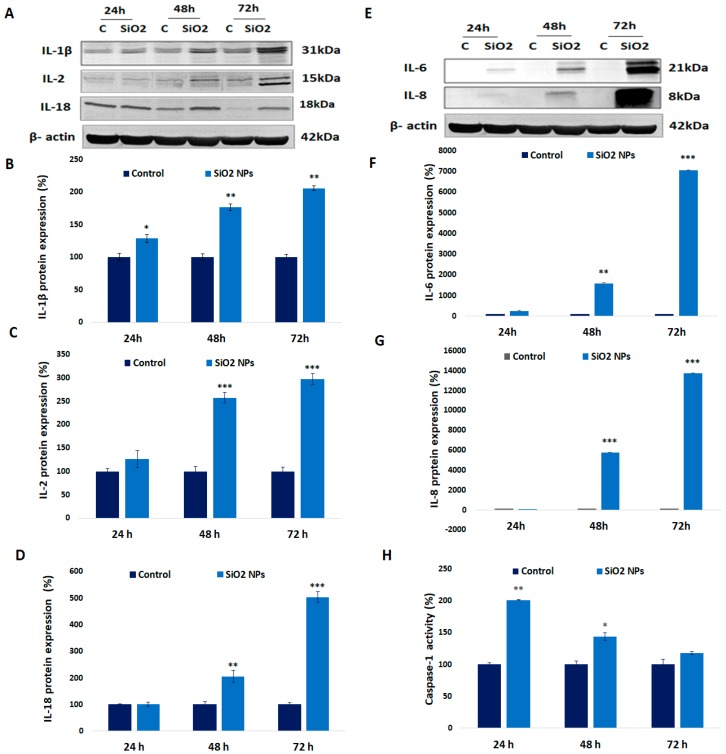
Inflammatory response in MRC-5 cells after SiO_2_ NPs exposure. The blot membranes of interleukins (**A**,**E**) were quantified and represented as the protein levels of interleukins (**B**) IL-1β, (**C**) IL-2, (**D**) IL-18, (**F**) IL-6, and (**G**) IL-8 after 24 h, 48 h, and 72 h of incubation with 62.5μg/ml SiO_2_. Activity of caspase-1 (**H**) was measured as described in Section 2.11. Values are mean SD (n = 3). * *p* < 0.05, ** *p* < 0.01, and *** *p* < 0.001.

The exposure of MRC-5 cells to 62.5 μg/mL of SiO_2_ NPs induced a time-dependent increase in IL-1β, IL-2, IL-6, IL-8, and IL-18 protein levels compared to control after 24 h, 48 h, and 72 h, respectively (Figure 8). In all of the cases, the maximum levels were reached after 72 h of exposure. Our study showed that SiO_2_ NPs induced increased levels of caspase-1 in MRC-5 cells (Figure 8). The level of caspase-1 significantly increased by 100%, 43%, and 17%, after 24 h, 48 h, and 72 h, respectively, compared to control. Caspase-1 is implicated in inflammation by cleavage of the IL-1β, IL-18, and IL-33 precursors.

### 3.9. Activity and Expression of MMPs in Human Lung Fibroblasts 

**Figure 9:**
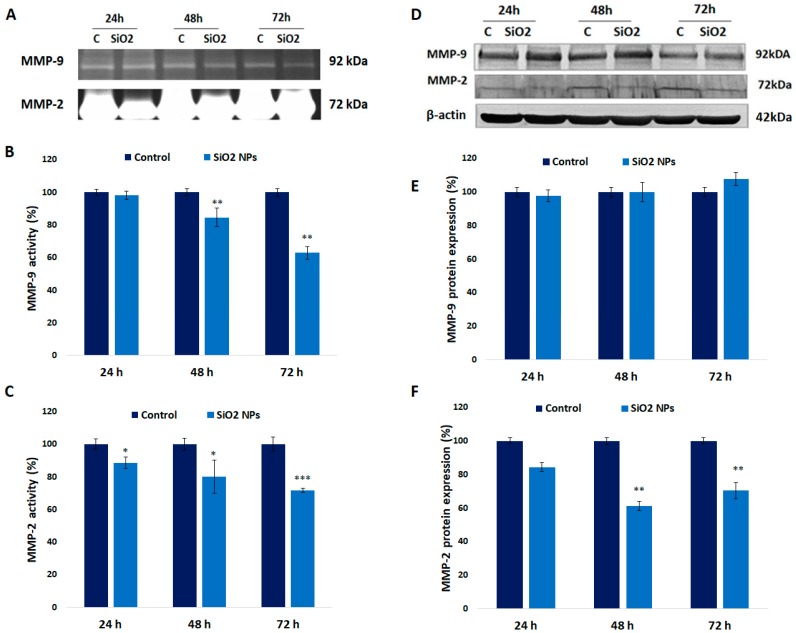
Enzymatic activity of MMPs in MRC-5 cells after 24 h, 48 h, and 72 h of exposure (**A**). Zymography analysis of MMP-9 (**B**) and MMP-2 (**C**). Western blotting analysis of MMP-9 and MMP-2 (**D**) in MRC-5 cells. Densitometric analysis of the protein bands of MMP-9 (**E**) and MMP-2 (**F**). Values are mean SD (n = 3). * *p* < 0.05, ** *p* < 0.01 and *** *p* < 0.001.

The expression of MMP-2 was diminished by 16%, 39%, and 30% after 24 h, 48 h, and 72 h, respectively, whereas its activity decreased significantly by 20% and 30% after 48 h and 72 h, respectively (Figure 9F,C). Furthermore, significant decreases were registered after 48 h and 72 h (by 15% and 27%) in MMP-9 activity, whereas its expression was not significantly modified up to 72 h compared to control (Figure 9B,E). 

## 4. Discussion

Among the different types of SiO_2_ NPs, we have selected to investigate in the present work the effect of silica NPs obtained by laser ablation due to several reasons. The reactive laser ablation has multiple advantages compared to other methods of obtaining ultrafine silica powders, such as magnetron sputtering, solution phase oxidation/reduction, thermolysis/laser pyrolysis, electrochemical etching (anodic oxidation), the microwave-assisted method, and atmospheric pressure plasma. It is considered a very clean method, because no chemical precursors that could contaminate the nanoparticles are used, it is controllable, has a very simple experimental setup, does not require high temperatures or pressures, and produces the desired powder stoichiometry. This type of technique can provide extremely fine powders, as the energy density and gas pressure are important parameters to decrease the particle size. 

Lung, due to its enormous surface, is a very important target for accidently inhaled SiO_2_ NPs, as well as for those used for drug and gene delivery by both systemic and local administration [28].

Our data indicated that these NPs with a hydrodynamic diameter of about 159 nm induced a low cytotoxicity in a time-dependent manner in MRC-5 lung fibroblasts (Figure S1), even if their primary size was less than 10 nm (Figure 1) [24]. However, due to their low dimensions, their aggregation tendency was important. These results are confirming those obtained by other research groups [29] and [28], who noticed that SiO_2_ NPs with sizes lower than 30 nm were more cytotoxic compared to those higher than 60 nm in human endothelial cells and lung epithelial cells, respectively. The same dependence, size versus toxicity, was noticed for silica nanoparticles in THP-1 derived macrophages, HaCaT keratinocytes and NRK-52E kidney cells [30].

The uptake of NPs occurs by two different mechanisms: pinocytosis, which involves the uptake of fluids and molecules within small vesicles, and phagocytosis, which includes the taking over of large particles [31]. There are several types of pinocytosis: macropinocytosis, clathrin-mediated endocytosis, caveolin-mediated endocytosis, clathrin-independent endocytosis, and caveolin-independent endocytosis [32]. However, not all cells can perform all types of endocytotic pathways due to their own physiological characteristics. The uptake of NPs into cells depends on their size, shape, and surface charge [33] and on biocorona [34] that is formed in the physiological environment on the NPs surface due to biomolecules’ association. 

The biocorona formed in the extracellular medium could influence the uptake mechanism of NPs by cells and their final subcellular location, resulting in different cellular responses [34].

Previously, it was proven that the uptake of SiO_2_ NPs in fibroblasts occurred by micropinocytosis, clathrin-mediated endocytosis, and caveolae-mediated endocytosis [35]. In other types of cells, clathrin-mediated endocytosis was the major pathway [30,36]. In our experiment, the increase in the number of lysosomes (Figure 2 and Figure 3) and autophagic vacuoles could be considered proof that endocytosis is the major uptake pathway of these NPs. 

SiO_2_ NPs consist of an amorphous network of tetrahedrally bonded Si–O moieties that present free negatively charged silanol moieties. The silanol oxygen is nucleophilic, and can attack the electrophilic carbonyl groups of the peptide bonds of proteins [37], affecting their tridimensional structure and consequently their function in biological structures. Other studies have suggested that amorphous SiO_2_ NPs could be harmful due to the silanol groups that are ionized at pH = 7 in a proportion between 5–20% [38] and could attract basic surfaces of proteins, disturbing their structures and functions. Furthermore, it was proven that amorphous SiO_2_ NPs synthesized through high-temperature methods were more toxic for erythrocytes, epithelial cells, and macrophage cells, compared to those obtained by low-temperature routes, which was probably due to important differences in the surface chemistry [39].

Previously [24], it was proven that this dose of NPs generated extracellular reactive oxygen species (ROS), which was probably due to the mechanical activation of cellular membrane NADPH (nicotinamide adenine dinucleotide phosphate reduced) oxidase and reasonably, superoxide anion penetrated cell membranes through the chloride channel-3 [40]. Alternatively, it was dismutated extracellularly, generating hydrogen peroxide that entered cells through aquaporin channels [41]. The late release of LDH in the culture media (Figure 4) could be probably explained by the capability of MRC-5 cells to counteract to a certain extend the oxidative stress generated by these NPs. 

Nuclear factor (erythroid-derived 2)-like 2 (Nrf-2) is an important transcription factor controlling cellular homeostasis in response to oxidative stress that drives the transcription of hundreds of genes implicated in different functions [42]. One of these mediates the transcription of components of the pentose phosphate pathway that generates NADPH, which is an important reducing molecule in the framework of the antioxidant system [43]. Cellular levels and the localization of Nrf-2 are strictly regulated by different mechanisms. One of these is mediated by the interaction with Kelch-like ECH (erythroid cell-derived protein with Cap ‘n’ Collar homology)-associated protein 1 (Keap1)–Cullin 3–Rbx1 complex. The human Keap 1 protein presents 27 cysteine residues, some of which can be oxidized in the presence of oxidizing and electrophilic agents, generating a conformational change. Due to this, its tridimensional structure is modified, and Keap 1 does not bind to Nrf2, which accumulates in the nucleus [44], where it interacts with small Maf (avian musculoaponeurotic fibrosarcoma oncogene homolog) proteins and binds to antioxidant responsive elements in the promoters of its target genes, followed by the initiation of transcription [45]. The human Keap 1 protein is involved in the transcription of several antioxidant genes that codify for: SOD, catalase, glutathione S-transferase, glutathione reductase, thioredoxin reductase, hem oxygenase-1, and others.

The induction of nitric oxide synthase is associated with oxidative stress and the activation of Nrf2 [46] and in our experimental conditions, in which the variation of NO concentration was directly correlated with the Nrf2 level (Figure 5).

Previously, it was proved [47] that the total SOD activity increased in time, and now, we noticed that the main contributor to this activity was MnSOD and not CuZn-SOD. MnSOD is present in the mitochondrial matrix and together with CuZn-SOD, which is located in the cytosol and mitochondrial intermembrane space, catalyzes the transformation of superoxide anion in hydrogen peroxide and molecular oxygen [48]. The mitochondrial isoenzyme maintains the levels of this superoxide at nanomolar concentration in physiological conditions, but also controls the cellular accumulation of hydrogen peroxide [49]. In our experiment, the variation of Nrf2 expression is indirectly correlated with MnSOD activity (Figure 6). So, initially due to the oxidative stress provoked by the interaction of SiO_2_ NPs with cells, Nrf2 expression was upregulated, and the transcription of the MnSOD gene probably increased, generating a moderate level of ROS that could control the equilibrium between survival and apoptosis through the activation of NF-kB [50]. As a result, a cross-link between Nrf2 and NF-kB could occur [51].

The Keap1/Cul3 complex could regulate both Nrf2 and NF-kB expression through ubiquitination, whereas Keap1 determines the ubiquitination of IKKß, which is a cytoplasmic NF-kB inhibitor [52]. It is probable that the active NF-kB is translocated in the nucleus, where it competes with Nrf2 as a co-activator of cyclic AMP-responsive element-binding (CREB)-binding protein and recruits histone deacetylase 3, causing a local hypoacetylation that prevents Nrf2 signaling [53]. In addition, Keap1 could interact with the p65 subunit of NF-kB, and due to this, the NF-kB signaling pathway decreases Nrf2 transcriptional activity [54].

In our experiments, the variation of Nrf2 expression (Figure 5A) was inversely proportional with that of NF-kB (Figure 5B), suggesting the regulation described above. 

TNF-α is a classical NF-kB-dependent proinflammatory cytokine [55] that has both autocrine and paracrine functions [56]. On the other hand, TNF-α in the active trimeric form binds to two different membrane bound receptors, TNFR1 and TNFR2, which are able to activate NF-kB. Also, the binding of NPs to toll-like receptors [57] could result in the activation of NF-kB and the upregulation of genes encoding inflammatory cytokines, including TNF-α [58]. The variation of TNF-α expression (Figure 7B) in this experimental setup is correlated with COX-2 (Figure 7A). Previously, it was observed that in human gingival fibroblasts, TNF-α induced PGE_2_ release and COX-2 mRNA upregulation [59]. 

Also, NF-kB targets genes involved in the generation of inflammation such as: pro-IL-1β [60], IL-2 [61], IL-6 [62], IL-8 [63], and IL-18 [61] as well as pro-caspase 1 [64]. Caspase 1 is a cysteine protease that is synthesized as a zymogen and becomes activated in the NLRP3 inflammasome. 

Previously, it was noticed that higher superoxide concentration inhibited caspase 1 by reversible oxidation and the glutathionylation of its redox-sensitive Cys 397 and Cys 362 residues [65]. In our recent paper [24], an increase of intracellular oxygen species (ROS) and a decrease of reduced glutathione (GSH) concentrations were observed in the same experimental setup. Probably due to the increase of ROS, the activity of caspase 1 decreased in a time-dependent manner, even if it remained higher than control up to 72 h of exposure.

The induction of pro-IL-1β (31 kDa) expression is followed by its processing done by caspase 1, in the framework of a multi-protein complex termed the NLRP3 inflammasome [66], which cleaves the N-terminal fragment of 116 amino acid residues in order to become mature [67] and secretes an active IL-1β molecule of 17 kDa. Similarly, IL-18, belonging to the same family of IL-1, is produced as a 24-kDa inactive precursor and activated by a limited proteolysis catalyzed by caspase 1 to a biologically active mature 18-kDa moiety. Probably, in the activation of IL-1β and IL-18, the caspase 1 and caspase 8-dependent pathways were coincident and due to this, the increase of these cytokines levels occurred even if the caspase 1 activity diminished up to 72 h [68] IL-1β plays an important role in fibrosis in vivo [67] as well as in vitro [69]. IL-18 has also profibrotic actions in vivo [70]. Furthermore, IL-1β could also stimulate COX-2, IL-6 [71], and IL-8 [72] production, as it was noticed before. IL-8 is a neutrophil chemotactic factor that in the context of lung might be involved in a variety of diseases [73], while IL-6 is synthesized in the initial stage of inflammation and has pleiotropic effects [74]. On the other hand, IL-2 is associated with skin and lung inflammation [75] and has anti-inflammatory effect [76].

As it can be seen in Figure 8, the variation of IL-1 β, IL-18, IL-6, and IL-8 expression had the same pattern, suggesting a direct correlation with the NF-kB level. Previously, in vivo studies with mice exposed to SiO_2_ NPs revealed the same results in lung cells [77] as ours.

Other modulators of inflammation are MMPs that are implicated in the degradation of the extracellular matrix either protecting against or contributing to pathology conditions in inflammatory processes. From a structural point of view, they are zinc-dependent endoproteinases that consist of a pro-peptide with a cysteine-switch motif, a catalytic metalloproteinase domain, a linker peptide of variable length, and a hemopexin domain [78]. 

TNF-α modulates the expression of MMP-2 and MMP-9 in pulmonary fibroblasts, but the upregulation of MMP-2 is much more important than that of MMP-9 [79].

In our experiment, the activity of MMP-2 decreased in a time-dependent manner (Figure 9A–C), being correlated with its protein expression (Figure 9D–F). Possibly, SiO_2_ NPs could penetrate into the nucleus, and due to their negative charge, bind to the positively charged histones leading to the reduction of MMP-2 gene transcription [80]. On the other hand, MMP-9 protein expression was not changed significantly in time, but its enzymatic activity decreased in a time-dependent manner. It is probable that SiO_2_ NPs could chelate the Zn ion from the active site of MMP-2 and MMP-9 at the low alkaline pH of their catalytic sites [81], as it was shown at higher pH values [82]. Our results differ from those of Dagonassat et al. [83], who demonstrated that amorphous SiO_2_ NPs upregulated MMP-2 and MMP-9 expression in mice lung tissue.

## 5. Conclusions

As far as we know, this is the first study regarding the inflammatory response induced by the exposure of MRC-5 lung fibroblasts to amorphous, stable SiO_2_ NPs synthetized by laser ablation. The incubation with negatively charged SiO_2_ NPs with 10-nm diameter generated the induction of proinflammatory markers and anti-inflammatory IL-2 by the upregulation of NF-kB, as well as a decrease of MMP activities. These effects are most probably due to the physicochemical characteristics of the studied NPs; being nucleophilic, the silanol oxygen can attack the electrophilic carbonyl groups of the peptide bonds of proteins and alter their tridimensional conformation and biological function. This inflammation and associated oxidative stress could determine the generation of a variety of potential outcomes at lung fibroblasts level. One of these could be represented by the deposition of increasing amounts of extracellular matrix components and the development of scar tissue, which is typical for fibrosis.

## Supplementary Materials

The following are available online at https://www.mdpi.com/1996-1944/12/7/1026/s1, Figure S1: The viability of MRC-5 human lung fibroblast cells exposed to SiO_2_ NPs, at different concentrations, for 24 h, 48 h, and 72 h. Values are calculated as means SD (n = 3) and are expressed as % from controls.
