# Supplementary material for: Amorphous Silica Nanoparticles Obtained by Laser Ablation Induce Inflammatory Response in Human Lung Fibroblasts
Source: Materials (Basel). 2019 Mar 28;12(7):1026. doi: 10.3390/ma12071026 (PMC6479987; doi:10.3390/ma12071026)
Supplement: Supplementary file 1 [file materials-12-01026-s001.pdf]

Supplemental material

Amorphous Silica Nanoparticles Obtained by Laser Ablation Induce Inflammatory Response in Human Lung Fibroblasts

Sorina Nicoleta Voicu [1,2], Mihaela Balas [1], Miruna Silvia Stan [1], Bogdan Trică [3], Andreea Iren Serban[4], Loredana Stanca[4], Anca Hermenean [5,6] and Anca Dinischiotu [1,*]

Cell viability was measured using an MTT (3-(4,5-dimethylthiazol-2-yl)-2,5-diphenyltetrazolium bromide, a tetrazole) assay.

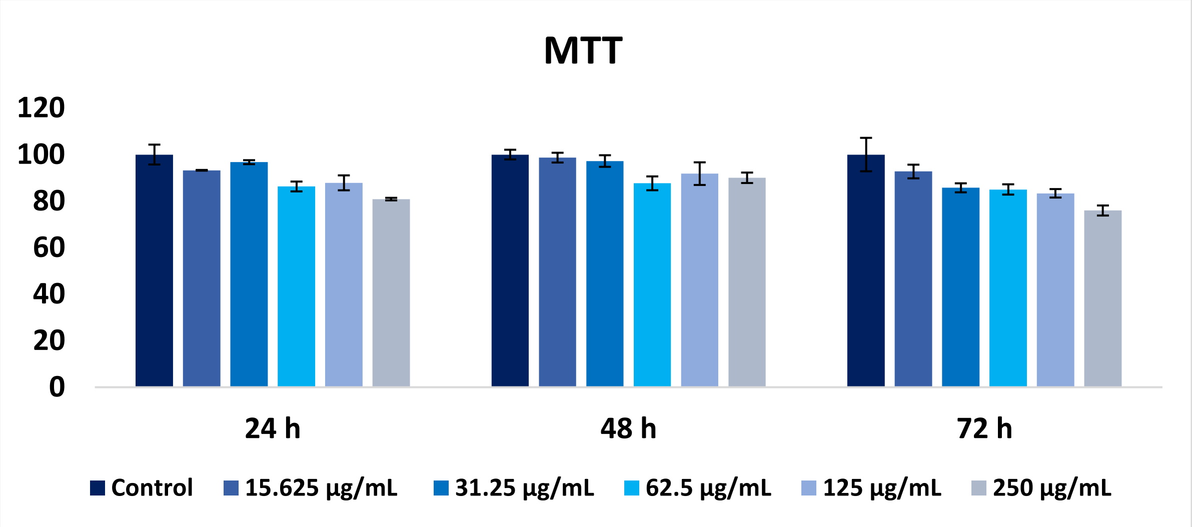

Figure S1. The viability of MRC-5 human lung fibroblast cells exposed to SiO_2 NPs, at different concentrations, for 24 h, 48 h, and 72 h. Values are calculated as means SD (n = 3) and are expressed as % from controls.
